# Gruel

**Published:** 1893-02

**Authors:** 


### GRUEL.

To make barley gruel, boil 4 oz. of pearl barley, or 1 teacupful in 3 cupfuls of water; boil it down to one quart. Strain and return to saucepan; grate into it a little cinnamon, if you like, and sweeten; add from one-half to three-quarters of a pint of fresh milk; warm up and use as wanted. For rice gruel take 2 tablespoonfuls of rice, 6 tablespoonfuls of cold water, 1 1-2 pints of new milk. Wash the rice and soak it in the cold water one hour. Put it into a double kettle with the milk and cook until the rice is well done and strain through a wire sieve. It can be sweetened if desired. Or a thin batter can be made of ground rice and milk, stirred into a generous pint of milk or water, and allowed to boil ten or fifteen minutes. In case of summer complaint brown the rice in the oven before cooking. To make gruel for the very delicate take a heaping teaspoonful of pearl tapioca, to one quart of water. Wash the tapioca and put it into the water cold, and let it come to the boiling point slowly. Let it boil gently until the tapioca is very soft. Strain it and add a little salt and cream if the patient can take it. This gruel is relished, and has proved beneficial where all other nourishment has been rejected. In case of bowel troubles, the addition of raisins in the proportion of a tablespoonful to a cup of milk cooked in the milk, is of value.

